# Thermomechanical Buckling Analysis of the E&P-FGM Beams Integrated by Nanocomposite Supports Immersed in a Hygrothermal Environment

**DOI:** 10.3390/molecules26216594

**Published:** 2021-10-30

**Authors:** Mohammad Khorasani, Luca Lampani, Rossana Dimitri, Francesco Tornabene

**Affiliations:** 1 Department of Basic and Applied Sciences for Engineering, Sapienza University, 00185 Rome, Italy; khorasani.1829460@studenti.uniroma1.it; 2 Department of Mechanical and Aerospace Engineering, Sapienza University, 00184 Rome, Italy; luca.lampani@uniroma1.it; 3 Department of Innovation Engineering, Università del Salento, 73100 Lecce, Italy; rossana.dimitri@unisalento.it

**Keywords:** CNTs, FGMs, high-order shear deformation theories, hygrothermal environment, thermomechanical buckling, sandwich beams

## Abstract

Due to the widespread use of sandwich structures in many industries and the importance of understanding their mechanical behavior, this paper studies the thermomechanical buckling behavior of sandwich beams with a functionally graded material (FGM) middle layer and two composite external layers. Both composite skins are made of Poly(methyl methacrylate) (PMMA) reinforced by carbon-nano-tubes (CNTs). The properties of the FGM core are predicted through an exponential-law and power-law theory (E&P), whereas an Eshelby–Mori–Tanaka (EMT) formulation is applied to capture the mechanical properties of the external layers. Moreover, different high-order displacement fields are combined with a virtual displacement approach to derive the governing equations of the problem, here solved analytically based on a Navier-type approximation. A parametric study is performed to check for the impact of different core materials and CNT concentrations inside the PMMA on the overall response of beams resting on a Pasternak substrate and subjected to a hygrothermal loading. This means that the sensitivity analysis accounts for different displacement fields, hygrothermal environments, and FGM theories, as a novel aspect of the present work. Our results could be replicated in a computational sense, and could be useful for design purposes in aerospace industries to increase the tolerance of target productions, such as aircraft bodies.

## 1. Introduction

FGM-based structures serve as bi-phase beams, plates and shells whose properties vary across their thickness or length continuously. Over the last century, FGMs have increasingly attracted the interest of the scientific community for their use in different high-tech industries due to their outstanding mechanical properties compared to single-phase materials and structures. Some natural types of FGMs are visible, such as bamboo trees, teeth, bones, and human skin, which have evolved to meet a specific requirement in humans and their environment. For the first time, Shen and Bever [1] considered graded material composites, despite their limited knowledge and a general lack of sophisticated fabrication equipment. Subsequently, Japanese scientists in 1984 [2] applied this technique to an aerospace project, due to their necessity to have a 10 mm thickness thermal barrier with an internal and external temperature of 1000 K and 2000 K, respectively. Fu et al. [3] also studied the thermoacoustic response of a porous FGM cylindrical plate with a random distribution of pores. Moreover, Duc and Cong [4] studied the vibrational behavior of FGM plates using a Runge–Kutta method, whose model was affected by thermomechanical coupled loading conditions. In another work, Shen [5] studied the thermomechanical post-buckling behavior of a simply-supported FGM plate equipped with piezoelectric fiber-reinforced composite patches for sensors, actuators, transducers and active damping devices. Zenkour [6] studied the static deflection response of an FGM plate in a hygrothermal environment, while discussing the response sensitivity to different environmental conditions. Transient analysis of porous FGM plates was also considered by Van et al. [7] in a nonlinear domain subjected to a coupled hygrothermal and mechanical loading condition. A post-buckling study of sandwich plates with FGM skins was performed by Kiani and Eslami [8] in a numerical sense, focusing on the effect of the power-law index, foundation parameters and imperfections of the overall response. In addition to the theoretical studies, many researchers have experimentally investigated the vibration and damping properties of nanoparticle-reinforced composite materials [9,10,11,12].

Besides FGMs, other composite nanomaterials have attracted the interest of engineering device production, with the rapid development of various analytical and computational models designed to simulate their behavior even in a nonlocal sense. In this context, Arshid et al. [13] studied the dynamic and static behavior of annular FG graphene nanoplatelets (FG-GNPs) reinforced nanocomposite with porosities, and applied modified strain gradient theory (MSGT) to account for size-dependent effects. In another work, Foroutan et al. [14] surveyed the nonlinear buckling and vibration behavior of imperfect FG carbon nanotubes reinforced composite (FG-CNTRC) cylindrical shells in a hygrothermal environment. Similarly, Arshid et al. [15] applied a 3D plate theory to study the vibrations of sandwich structures with a honeycomb core and GNP-reinforced epoxy face sheets, as commonly found in many electric devices. Safaei [16] developed a generalized vibration model including the possible presence of porosity within sandwich structures (both in core and skins) immersed in a Pasternak foundation. Among the recent scientific literature, several theoretical and numerical methods have been proposed to solve complicated structural problems, involving advanced composite materials. Moradi-Dastjerdi et al. [17] applied a higher-order theory and the Eshelby–Mori–Tanaka (EMT) approach to assess the buckling behavior of a sandwich plate made of a CNTRC porous core with external piezoelectric face sheets, while assuming different CNTs’ agglomerations within the material. In a similar direction, the authors of Refs. [18,19] proposed a refined higher-order theory to evaluate the CNTs’ agglomeration impact on the vibration control and stiffness of nanocomposite sandwich beams, respectively. In addition, further efforts in this direction have been performed by other scholars [20,21,22,23,24,25,26,27,28,29,30,31,32,33,34,35,36,37,38,39,40,41,42,43,44,45,46,47,48,49]. 

Motivated by the aforementioned studies, this paper aims to further contribute to the thermomechanical response of sandwich beams including an exponential-law/power-law-based functionally graded core (E&P-FGC) integrated by two CNTRC layers. A unified framework was originally proposed to account for different high-order kinematic assumptions for the systematic study of sandwich structures resting on an elastic Pasternak substrate, under coupled hygrothermal loading conditions. The governing equations of the problem are determined from the virtual displacement principle, accounting for various beam theories and CNTs’ agglomeration effects. A Navier-type procedure was, thus, proposed to solve the problem analytically, whose results, based on different E&P-FGM relations and thermomechanical conditions could be very useful for further computational investigations on this topic, even from a practical design standpoint.

## 2. Analytical Model

**Figure 1:**
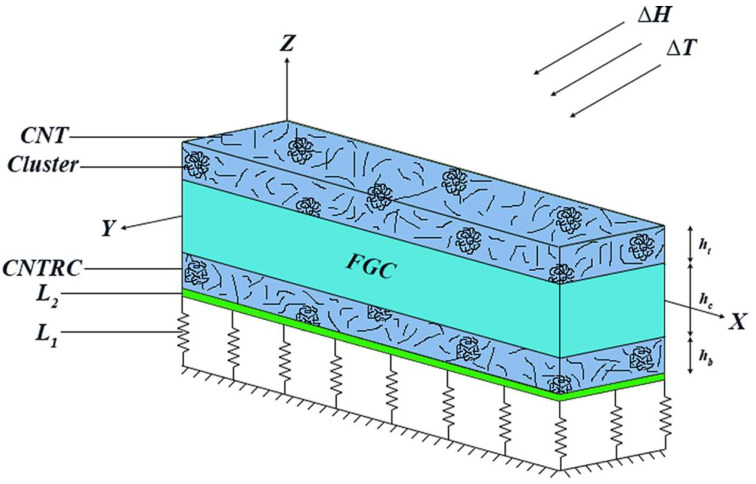
Sandwich beam model.

Let us consider a three-layer sandwich beam with length *a* and thickness *h*. As shown in Figure 1, the total thickness is the assemblage of an FGC, and two external (top and bottom) CNTRC skins, with thickness *h_c_*, *h_t_*, and *h_b_*, respectively. The model is referred to as the cartesian coordinate system (*x*, *y*, *z*), and it is located at the midplane of the model, as depicted in Figure 1. The whole structure is embedded in an elastic Pasternak substrate, and it is subjected to a variable hygrothermal surrounding condition.

For this sandwich structure, we propose and compare a thermomechanical model based on four different kinematic assumptions, namely, the third-order shear deformation theory (TSDT), the hyperbolic shear deformation theory (HSDT), the sinusoidal shear deformation theory (SSDT) and the exponential shear deformation theory (ESDT). Based on these kinematic assumptions, the displacement field for an arbitrary point in the mid surface is defined as [20,21].

(1) $$U\left(x,z\right)=u\left(x\right)-z\frac{\partial w\left(x\right)}{\partial x}+\left[\begin{array}{cc} z-\frac{4z^{3}}{3h^{2}} & TSDT\\ h\sinh\left(\frac{z}{h}\right)-z\cosh\left(\frac{1}{2}\right) & HSDT\\ \frac{h}{\pi }\sin\left(\frac{\pi z}{h}\right) & SSDT\\ z\exp\left(-2{\left(\frac{z}{h}\right)}^{2}\right) & ESDT \end{array}\right]u_{1}\left(x\right),\;V\left(x,z\right)=0,\;W\left(x,z\right)=w\left(x\right)$$

where *u* and *w* refer to the longitudinal and transverse displacement components of the mid surface. For simplicity reasons, Equation (1) can be rewritten in a more compact notation by introducing a general function *Q*(*z*) to define the terms in the square brackets, depending on the selected kinematic theory. Thus, by simply changing the definition of *Q*(*z*), we are able to switch among different displacement assumptions.

The kinematic relations between the strain and displacement fields are, thus, expressed as [22]:

(2) $$\varepsilon_{xx}^{c,t,b}=\frac{\partial U}{\partial x},\;\gamma_{xz}^{c,t,b}=\frac{\partial U}{\partial z}+\frac{\partial W}{\partial x}$$

where superscripts *c*, *t*, and *b* refer to the core, top, and bottom layer.

For every single layer of the sandwich structure, we refer to the following constitutive relations [23,24]:

(3) $$\sigma_{xx}^{c,t,b}=A_{11}^{c,t,b}\varepsilon_{xx}-\alpha_{xx}^{c,t,b}\;\;\Delta T-\beta_{xx}^{c,t,b}\;\;\Delta H,\;\tau_{xz}^{c,t,b}=A_{55}^{c,t,b}\gamma_{xz}$$

where *A* refers to the elastic constants, *β* and *α* denote the moisture and thermal expansion coefficients, respectively, Δ*T* and Δ*H* stand for the thermal and moisture variation, respectively, defined as $\Delta T=T\left(z\right)-T_{0}$ and $\Delta H=H\left(z\right)-H_{0}$. Moreover, *T*_0_ and *H*_0_ denote the ambient temperature and moisture, here kept equal to 293 K and 0.1 wt%H_2_O, respectively. Furthermore, *T*(*z*) and *H*(*z*) represent a linear temperature and moisture variation across the thickness direction of the beam [25]:

(4) $$T(z)=T_{b}+\Delta T_{t\;b}\left(\frac{h+2z}{2h}\right),\;H(z)=H_{b}+\Delta H_{t\;b}\left(\frac{h+2z}{2h}\right)$$

where subscripts *t* and *b* refer to the top and bottom surface of the model. At the same time, Δ*T_tb_* and Δ*H_tb_* stand for the thermal and moisture variation between the top and bottom sides, namely, Δ*T_tb_ = T_t_ − T_b_* and Δ*H_tb_ = H_t_ − H_b_*.

**Figure 2:**
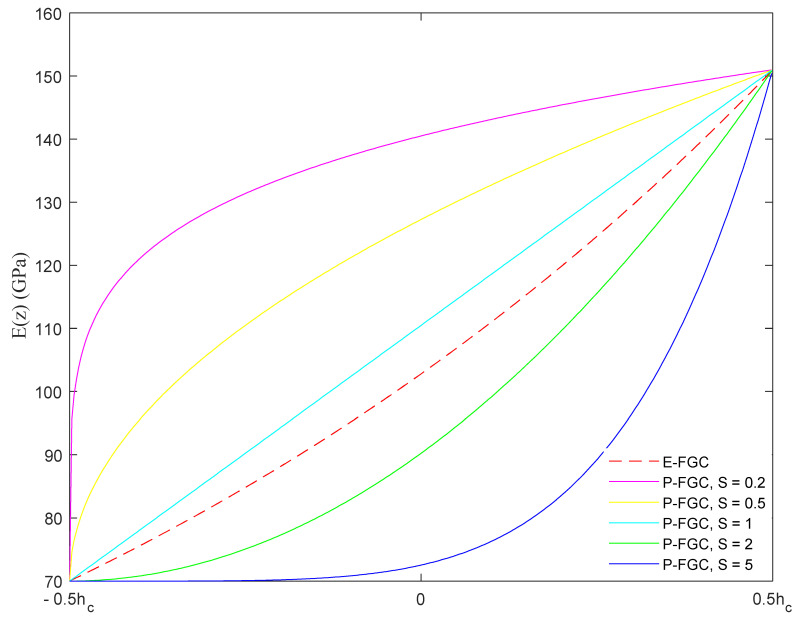
Variation of the elasticity modulus with the core thickness based on the E-FGC and P-FGC models.

The core layer of the selected sandwich beam is made up of FGMs, which means that the top and bottom surfaces of the FGC are made of pure ceramic and pure metal, whereas from the bottom to the top surfaces, the volume fraction of the metal and ceramic phases vary, keeping fixed *V_c_* + *V_m_* = 1. In what follows, we consider two different variations of material properties throughout the thickness of the FGC, evaluated here comparatively for their impact on the thermomechanical buckling behavior of the whole structure. Based on the P-FGC and E-FGC, the mechanical properties of the FGC are defined as:

(5) $$J^{c}(z)=J_{m}\exp\left(\log\left(\frac{J_{c}}{J_{m}}\right)\left(\frac{h_{c}+2z}{2h_{c}}\right)\right)\;\;\;\;\;\;\;\;\;\;\;\;\;\;\;\;\;\;E-FGC\;J^{c}(z)=(J_{c}-J_{m})\;\;{\left(\frac{h_{c}+2z}{2h_{c}}\right)}^{S}+J_{m},\;\;\;\;\;\;\;\;\;\;\;\;\;\;\;\;\;\;\;\;P-FGC$$

where subscripts *c* and *m* denote the ceramic and metallic properties, respectively. Moreover, *J* is a general notation that defines the arbitrary mechanical property, as the elasticity modulus *E*, Poisson’s ratio *ν*, density *ρ*, *β* and *α*. In the case of P-FGC, *S* is the power-law index. Allocating a zero value to *S*, the FGC reverts to a fully ceramic layer; when S=∞ the core layer is fully metallic. Based on the two-fold definition of *J^c^*(*z*) in Equation (5), the elasticity modulus varies in the FGC thickness as plotted in Figure 2. 

The sandwich beam system includes two CNTRC faces, with increased overall stiffness. Both external skins are assumed to be completely bonded to the FGC without any possible interlayer slip. The presence of CNTs within the PMMA matrix plays an efficient role in the general improvement of the mechanical properties. Due to the high aspect ratio of CNTs, their bending and bundling within the matrix phase and clusters formation seem to be almost predictable. In this paper, we consider the effect of the CNTs’ agglomeration on the overall mechanical response, based on an EMT scheme [26]. Based on this approach, the bulk modulus *K* and shear modulus *G* inside and outside the clusters can be defined as [26,27]:

(6) $$K_{in}^{t,b}=\frac{3K_{p}(\mu -V_{r}\eta +V_{r}\eta \alpha_{r})+(\delta_{r}-3K_{p}\alpha_{r})\;V_{r}\eta }{3(\mu -V_{r}\eta +V_{r}\eta \alpha_{r})},$$

(7) $$G_{in}^{t,b}=\frac{2G_{p}(\mu -V_{r}\eta +V_{r}\eta \beta_{r})+(\eta_{r}-2G_{p}\beta_{r})\;V_{r}\eta }{2(\mu -V_{r}\eta +V_{r}\eta \beta_{r})},$$

(8) $$K_{out}^{t,b}=\frac{3K_{p}((1-\mu )-V_{r}(1-\eta )+V_{r}(1-\eta )\alpha_{r})+(\delta_{r}-3K_{p}\alpha_{r})\;V_{r}(1-\eta )}{3((1-\mu )-V_{r}(1-\eta )+V_{r}(1-\eta )\alpha_{r})},$$

(9) $$G_{out}^{t,b}=\frac{2G_{p}((1-\mu )-V_{r}(1-\eta )+V_{r}(1-\eta )\beta_{r})+(\eta_{r}-2G_{p}\beta_{r})\;V_{r}(1-\eta )}{2((1-\mu )-V_{r}(1-\eta )+V_{r}(1-\eta )\beta_{r})}$$

where subscripts *p* and *r* refer to the polymeric matrix and CNTs. 

Furthermore, *μ* and *η* denote the CNTs’ agglomeration region volume fraction and the cluster’s CNTs’ volume fraction. It should be mentioned that the value of *μ* can be lower or equal to *η*. Regarding the definition and value assumed by *μ* and *η*, some possible cases can occur, as detailed in Table 1.

**Figure 3:**
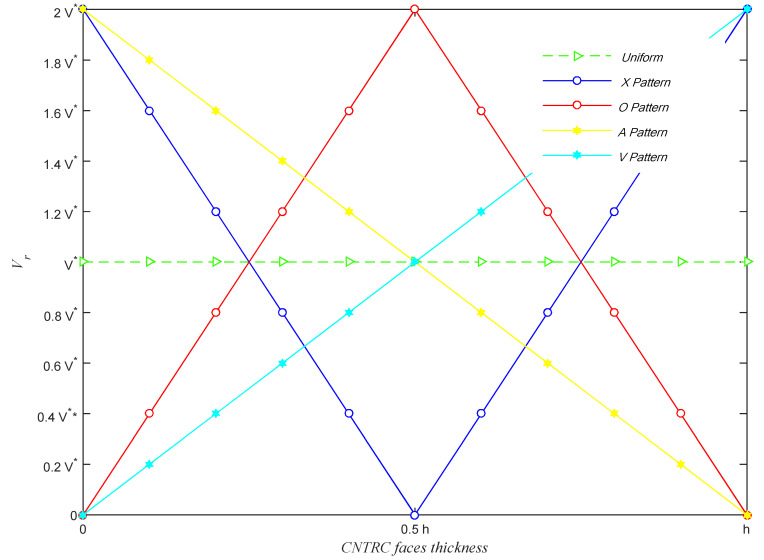
Variation of the CNTs’ volume fraction versus the CNTRC skin thickness for different types of CNTs’ patterns.

In order to capture the impact of different CNT distribution patterns inside the composite faces, the following equations can be used to define the CNT volume fraction inside the composite skins [28]:

(10) $$V_{r}=\{\begin{array}{cc} V^{*} & U\\ \frac{4}{h_{t,\;\;b}}\left|z\mp 0.5(h_{c}+h_{t,\;\;b})\right|V^{*} & FG-X\\ \left(\frac{2h_{t,\;\;b}-4}{h_{t,\;\;b}}\right)\left|z\mp 0.5(h_{c}+h_{t,\;\;b})\right|V^{*} & FG-O\\ \left(\frac{h_{t,\;\;b}-2}{h_{t,\;\;b}}\right)(z\mp 0.5(h_{c}+h_{t,\;\;b}))\;V^{*} & FG-A\\ \left(\frac{h_{t,\;\;b}+2}{h_{t,\;\;b}}\right)(z\mp 0.5(h_{c}+h_{t,\;\;b}))\;V^{*} & FG-V \end{array}$$

where V* refers to the weight fraction of CNTs. In Figure 3 we show the variation of the CNTs’ volume fraction versus the composite layer thickness for different types of CNTs’ distributions. Also, it should be mentioned that V* is the CNTs’ volume fraction in the case of their uniform dispersion.

In addition, the terms *α_r_*, *β_r_*, *η_r_* and *δ_r_* can be written as follows [17]:

(11) $$\alpha_{r}=\frac{3(K_{p}+G_{p})+k_{r}-l_{r}}{3(G_{p}+k_{r})},$$

(12) $$\beta_{r}=\frac{4G_{p}+2k_{r}+l_{r}}{15(G_{p}+k_{r})}+\frac{4G_{p}}{5(G_{p}+p_{r})}+\frac{2\left(G_{p}(3K_{p}+G_{p})+G_{p}(3K_{p}+7G_{p})\right)}{5G_{p}(3K_{p}+G_{p})+5m_{r}(3K_{p}+7G_{p})},$$

(13) $$\begin{array}{l} \eta_{r}=\frac{2(n_{r}-l_{r})}{15}+\frac{8G_{p}m_{r}(3K_{p}+4G_{p})}{15K_{p}(G_{p}+m_{r})+5G_{p}(G_{p}+7m_{r})}+\frac{2(k_{r}-l_{r})(2G_{p}+l_{r})}{15(G_{p}+k_{r})}\\ +\frac{8G_{p}p_{r}}{5(G_{p}+p_{r})}, \end{array}$$

(14) $$\delta_{r}=\frac{n_{r}+2l_{r}}{3}+\frac{\left(2k_{r}+l_{r})(3K_{p}+2G_{p}-l_{r}\right)}{3(G_{p}+k_{r})}$$

where *k_r_*, *l_r_*, *m_r_*, *p_r_* and *n_r_* denote the CNTs’ elastic Hill’s constants which are varied for different types of CNTs with respect to their chiral index value. In the current study, such constants are defined for single-walled carbon nanofillers (SWCNTs) with a chirality index equal to 10 [29].

Thus, the bulk modulus and shear modulus of the external skins can be obtained as [30]:

(15) $$K_{n}^{t,b}(z)=K_{out}^{t,b}+\frac{\mu \;(K_{in}^{t,b}-K_{out}^{t,b})}{1+P^{t,b}(1-\mu )\left(\frac{K_{in}^{t,b}-K_{out}^{t,b}}{K_{out}^{t,b}}\right)},$$

(16) $$G_{n}^{t,b}(z)=G_{out}^{t,b}+\frac{\mu (G_{in}^{t,b}-G_{out}^{t,b})}{1+I^{\;t,b}(1-\mu )\left(\frac{G_{in}^{t,b}-G_{out}^{t,b}}{G_{out}^{t,b}}\right)}$$

in which subscript *n* denotes the nanocomposite faces, and:

(17) $$P^{\;t,b}=\frac{1+\nu_{out}^{t,b}}{3(1-\nu_{out}^{t,b})}\;I^{\;t,b}=\frac{8-10\nu_{out}^{t,b}}{15(1-\nu_{out}^{t,b})}$$

where,

(18) $$\nu_{out}^{t,b}=\frac{3K_{out}^{t,b}-2G_{out}^{t,b}}{6K_{out}^{t,b}+2G_{out}^{t,b}}$$

The mechanical properties of the composite faces including *E*, *ν*, *ρ*, *α* and *β* can be addressed as [17,31]:

(19) $$E^{t,b}(z)=9K_{n}^{t,b}G_{n}^{t,b}/\left(3K_{n}^{t,b}+G_{n}^{t,b}\right),$$

(20) $$\nu^{\;t,b}(z)=\left(3K_{n}^{t,b}-2G_{n}^{t,b}\right)/\left(6K_{n}^{t,b}+2G_{n}^{t,b}\right),$$

(21) $$\rho^{t,b}(z)=(\rho_{r}^{t,b}-\rho_{p}^{t,b})\;V_{r}+\rho_{p}^{t,b},$$

(22) $$\alpha^{\;t,b}(z)={\left(\frac{E+4\nu K_{n}(1+\nu )}{E+4K_{n}{\left(1+\nu \right)}^{2}}\right)}^{\;\;t,b}\left(\frac{V_{r}E_{11}^{CNT}\alpha_{11}^{CNT}+(1-V_{r})E_{p}\alpha_{p}}{V_{r}E_{11}^{CNT}+(1-V_{r})E_{p}}\right)$$

(23) $$\beta^{\;t,b}(z)={\left(\frac{E+4\nu K_{n}(1+\nu )}{E+4K_{n}{\left(1+\nu \right)}^{2}}\right)}^{\;\;t,b}\left(\frac{V_{r}E_{11}^{CNT}\beta_{11}^{CNT}+(1-V_{r})E_{p}\beta_{p}}{V_{r}E_{11}^{CNT}+(1-V_{r})E_{p}}\right)$$

Finally, the elastic constants for the external layers can be defined as [32]:

(24) $$\begin{array}{l} A_{11}^{c,t,b}(z)=\frac{E^{c,t,b}(z)}{1-{\left(\nu^{c,t,b}(z)\right)}^{2}}\\ A_{55}^{c,t,b}(z)=\frac{E^{c,t,b}(z)}{2(1+\nu^{c,t,b}(z))} \end{array}$$

## 3. Governing Equations

We now apply the virtual work principle to determine the governing equations of the problem [15]:

(25) δ(Ω−Ψ)=0

where Ω is the strain energy and Ψ is the external work.

The classical strain energy for a sandwich beam is defined as [33]:

(26) $$\Omega =\frac{1}{2}\int_{x}\int_{z}{\left(\sigma_{xx}\varepsilon_{x}+\tau_{xz}\gamma_{xz}\right)}^{c,t,b}dzdx$$

The external work includes three terms, namely, the thermal force, the external force related to humidity and the elastic substrate force, defined as follows [34]:

(27) $$F^{T}=\int_{z}A_{11}^{\;c,t,b}\alpha^{c,t,b}\;\Delta Tdz,\;F^{H}=\int_{z}A_{11}^{\;c,t,b}\beta^{c,t,b}\;\Delta Hdz,\;F^{f}=L_{1}w-L_{2}\frac{\partial^{2}w}{\partial x^{2}}$$

where *L*_1_ is the spring constant and *L*_2_ stands for the shear layer constant. The total external work reads as follows [35]:

(28) $$\Psi =\frac{1}{2}\int_{x}\left((F^{T}+F^{H}){\left(\frac{\partial w}{\partial x}\right)}^{2}-F^{f}w\right)dx$$

By substitution of Equations (26) and (28) into Equation (25), after mathematical manipulation, we obtain the following governing equations:

(29) $$\delta u:-\frac{\partial }{\partial x}\left(A_{0}\frac{\partial u}{\partial x}-A_{1}\frac{\partial^{2}w}{\partial x^{2}}+A_{3}\frac{\partial u_{1}}{\partial x}\right)=0,$$

(30) $$\delta u_{1}:-\frac{\partial }{\partial x}\left(A_{3}\frac{\partial u}{\partial x}+A_{4}\frac{\partial u_{1}}{\partial x}-A_{5}\frac{\partial^{2}w}{\partial x^{2}}\right)+A_{6}u_{1}=0,$$

(31) $$\delta w:-\frac{\partial^{2}}{\partial x^{2}}\left(A_{1}\frac{\partial u}{\partial x}-A_{2}\frac{\partial^{2}w}{\partial x^{2}}+A_{5}\frac{\partial u_{1}}{\partial x}\right)-L_{2}\frac{\partial^{2}w}{\partial x^{2}}+L_{1}w-F^{T}\frac{\partial^{2}w}{\partial x^{2}}-F^{H}\frac{\partial^{2}w}{\partial x^{2}}=0$$

where,

(32) $$\left[\begin{array}{c} A_{0}\\ A_{1}\\ A_{2}\\ A_{3}\\ A_{4}\\ A_{5} \end{array}\right]=\;\;\int_{z}A_{11}^{c,t,b}\left[\begin{array}{c} 1\\ z\\ z^{2}\\ Q(z)\\ Q^{2}(z)\\ zQ(z) \end{array}\right]dz\;A_{6}=\int_{z}A_{55}^{c,t,b}{\left(\frac{\partial Q(z)}{\partial z}\right)}^{2}dz\;\;$$

## 4. Analytical Solution Procedure

Equations (29)–(31) are solved here analytically by means of a Navier-type procedure. Based on this technique, we use the following expressions to define the displacement components, which satisfy simply supported boundary conditions [36]:

(33) $$\begin{array}{c} u(x)=\overset{\infty }{\underset{m=1}{\Sigma }}U\cos(\frac{m\pi }{a}x),\\ u_{1}(x)=\overset{\infty }{\underset{m=1}{\Sigma }}U_{1}\cos(\frac{m\pi }{a}x),\\ w(x)=\overset{\infty }{\underset{m=1}{\Sigma }}W\sin(\frac{m\pi }{a}x), \end{array}$$

where, *U*, *U*_1,_ and *W* serve as unknown coefficients; *m* denotes the mode number along the ESB length. By placing Equation (33) into Equations (29)–(31), after mathematical manipulation, the governing equations are easily solved.

## 5. Numerical Results and Discussions

In this section, we perform various numerical examples to demonstrate the accuracy of the formulation proposed herein, to study the critical buckling load of an Euler–Bernoulli beam (EBB) and Timoshenko beam (TB), for different values of power-law exponent and geometrical characteristics. Our results are compared to predictions by Li and Batra [37], as summarized in Table 2, with an acceptable agreement for the geometrical length-to-thickness ratios *a/h* = 5 and 10, and for all values of *S*. This confirms the reliability of the proposed formulation to handle such a topic. 

A systematic analysis is repeated, by assuming different input material and geometrical properties in the model, as listed in Table 3, Table 4 and Table 5, in line with Refs. [17,38], in which the term N* stands for the nondimensional critical buckling load.

**Figure 4:**
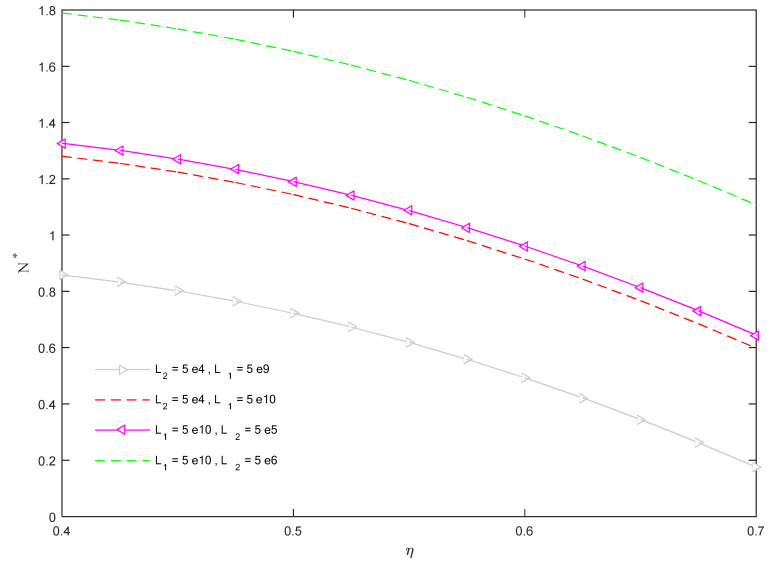
Dimensionless critical buckling load versus CNTs’ volume fraction within clusters for different foundation parameters.

In Figure 4, we plot the variation of the dimensionless critical buckling load N* vs. the CNTs’ volume fraction inside clusters, *η*, while setting different combinations of the foundation parameters *L*_1_, *L*_2_.

Based on results in this figure, CNTs’ inflation inside some concentrated regions (i.e., an increased value *η*) yields a reduced critical buckling load because of the stiffness reduction in the whole structure, in line with the experimental findings by Pan et al. [11]. On the other hand, by increasing the foundation parameters *L*_1_, *L*_2_, the critical buckling load is enhanced because of an increased stiffness configuration induced on the structure. 

**Figure 5:**
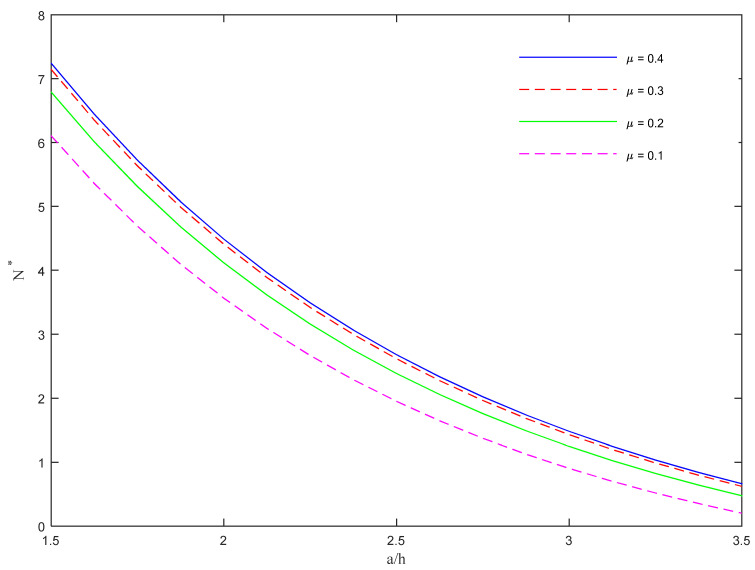
Dimensionless critical buckling load versus rational *a*/*h* for different values of *µ*.

As also plotted in Figure 5, the model is very sensitive to the rational length *a*/*h* and to the CNTs’ agglomeration region *µ*, with a monotonic reduction of N* for an increased *a*/*h* ratio, along with a gradual reduction of N* for a reduced level of *µ*, under a fixed value of *a*/*h*. This means that a decreased agglomeration region of CNTs causes a stiffness reduction of the structure, which can buckle more easily.

**Figure 6:**
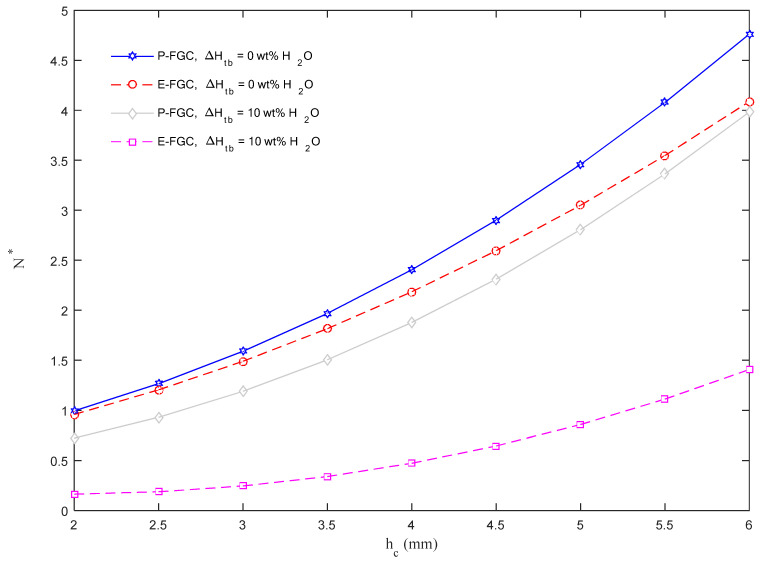
Dimensionless critical buckling load versus the core thickness for different moisture variations, based on E&P-FGC models.

The study considers the effect of different top–bottom moisture variations ΔH_tb_ based on the E&P-FGC models, while varying the core thickness *h_c_*, as represented in Figure 6. Based on the plots in Figure 6, it is visible that the application of a P-FGC model obtains higher values of the buckling load and structural stiffness compared to an E-FGC model, for a fixed value of ΔH_tb_. As also expected, an increased level of humidity in the surrounding environment has a destructive effect on the mechanical properties of the structure with a consecutive reduction of N* under the same assumption of *h_c_*. This mechanical decay for different humidity conditions represents a key aspect for many aerospace and marine applications, as well as nanostructures like generators and sensors. At the same time, an increased core thickness makes the structure stiffer, thus leading to a monotonic increase in the buckling load for each selected model and moisture variation. 

**Figure 7:**
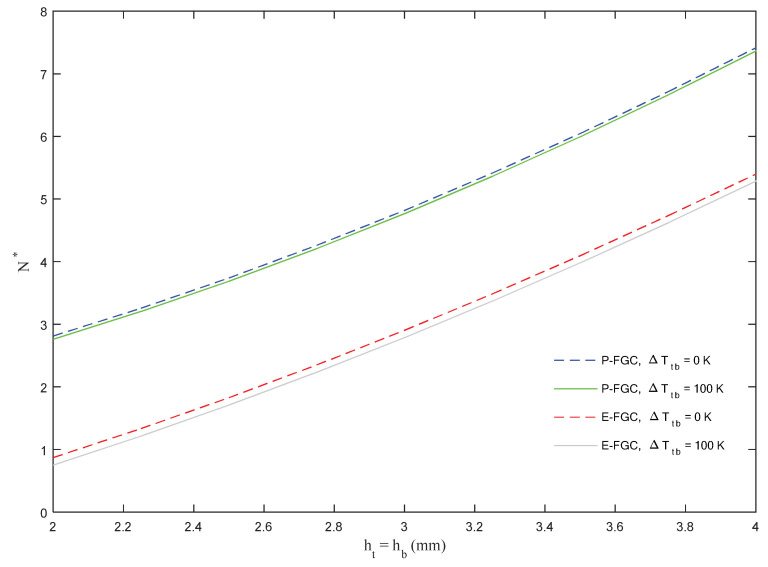
Dimensionless critical buckling load versus skin thickness for different thermal variations, based on E&P-FGC models.

We now repeat a similar investigation based on the E&P-FGC models, while checking for the sensitivity of the response to the thermal variation Δ*T_tb_* among the top and bottom sides of the sandwich structure together with a varying thickness of the external skins (see Figure 7). Based on the plots in this figure, an increased thickness of the top and bottom sides enhances the overall stiffness of the structure together with its buckling load, independently of the selected model and thermal variation. Moreover, for each selected model, the results are almost unaffected by the thermal variation Δ*T_tb_*, where a P-FGC model always obtains higher values of the buckling load with respect to an E-FGC model.

**Figure 8:**
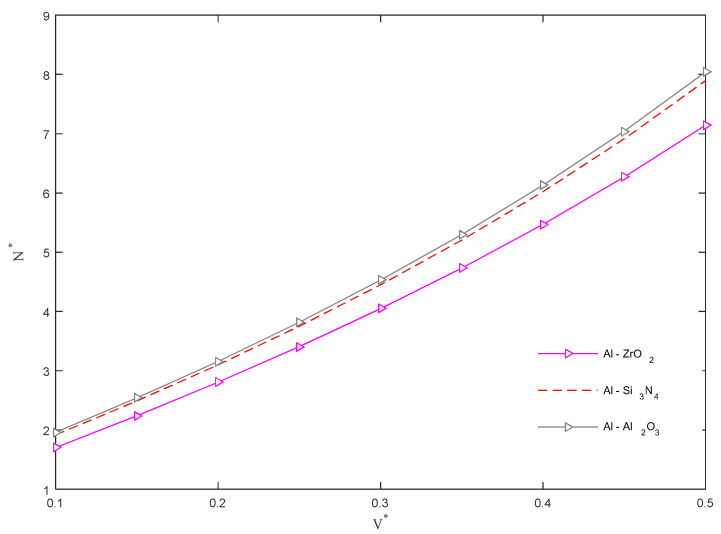
Dimensionless critical buckling load versus CNT volume fraction for different FGC material constituents.

Figure 8, instead, aims at evaluating the sensitivity of N* for different CNT volume fractions V* and different FGC material constituents. The monotonic increase in each curve for an increased level of V* demonstrates the beneficial effect of such a quantity on the overall buckling response of the structure. It was also found that the use of the Al in conjunction with Al_2_O_3_, Si_3_N_4_ and ZrO_2_ as FGC material constituents, obtains higher magnitudes of the buckling load.

**Figure 9:**
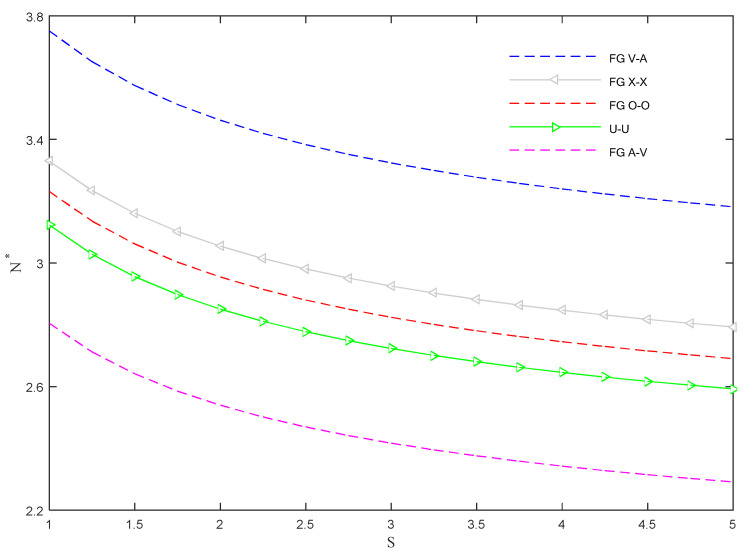
Dimensionless critical buckling load versus power-law index for different CNTs’ distribution patterns within the CNTRC skins.

As visible in Figure 9, an enhanced power-law index causes a general reduction of dimensionless critical buckling. Among different possibilities of the CNTs’ distributions, the FGA-V and FGV-A distributions yield the lowest and highest magnitudes of the buckling load, respectively, where other types of distribution yield intermediate predictions. 

**Figure 10:**
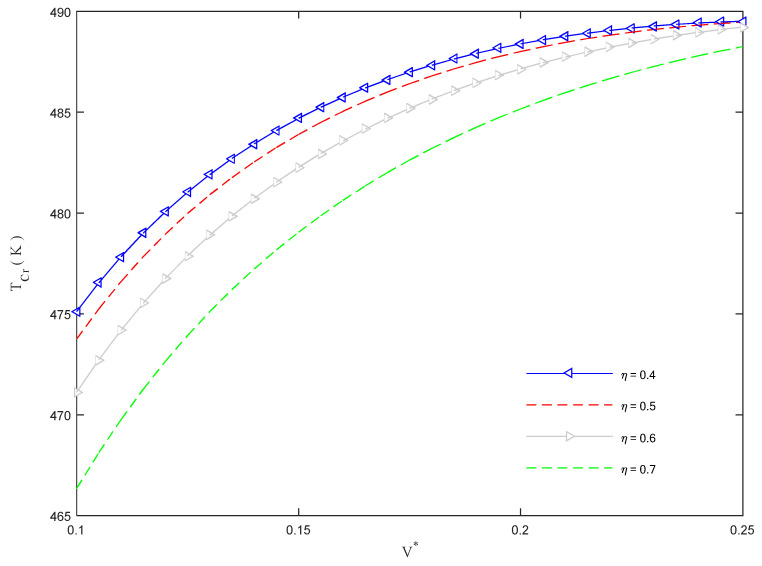
Critical buckling temperature versus CNTs’ volume fractions for different values of CNTs’ volume fractions inside the clusters.

Figure 10 displays the critical buckling temperature against the volume fraction of CNTs for different CNTs’ volume fractions, *η*. As visible in this figure, an increased value of V* improves the critical buckling temperature, especially for a reduced level of *η*.

**Figure 11:**
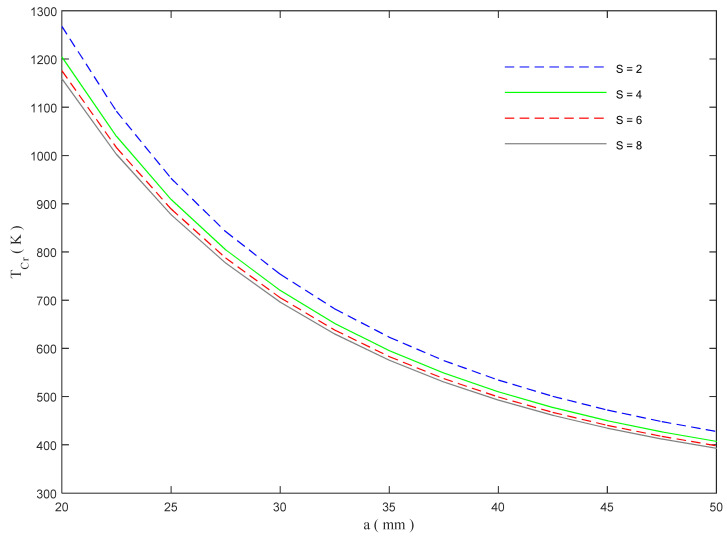
Critical buckling temperature versus the beam length, for different power-law indexes.

At the same time, the critical buckling temperature seems to reduce for an increased length of the beam, as plotted in Figure 11, for different values of the power-law index *S*. Moreover, the overall thermal response of the structure, seems to be slightly affected by *S*, whose increase produces a meaningless reduction in *T_cr_* under the same geometrical length.

**Figure 12:**
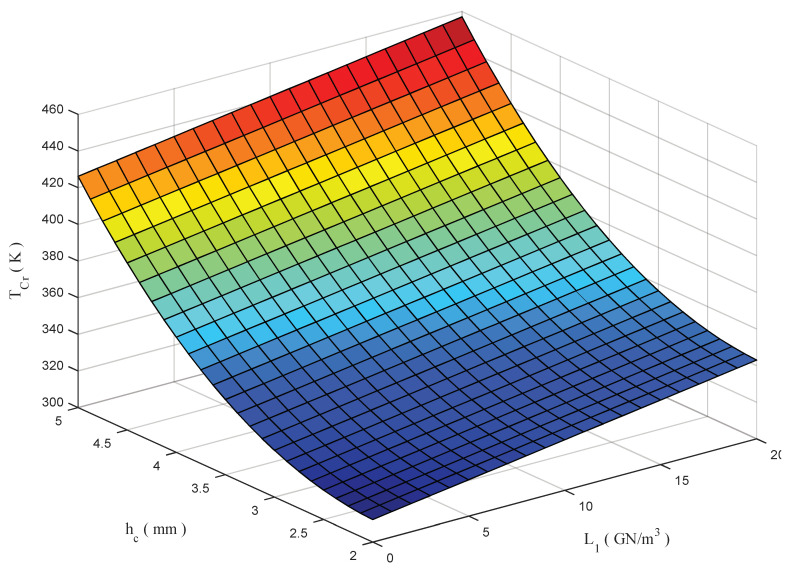
Double effect of core thickness and foundation spring parameter on the critical buckling temperature of a sandwich beam.

The final 3D plot in Figure 12 shows the double variation of *T_cr_* with the core thickness *h_c_* and substrate elastic constant L_1_. Note that the double increase in h_c_ and L_1_ produce the highest value of *T_c_*. The opposite effect is obtained for a simultaneous decrease in both parameters, which corresponds to the most serious thermal buckling condition. 

Finally, in Table 6, we summarize the impact of different beam theories on the dimensionless critical buckling load, while considering different FGC models and CNT volume fractions, in a numerical sense. It seems that TSDT and HSDT provide the highest and lowest predictions, respectively, whereas SSDT and ESDT always produce intermediate results. It is also found that P-FGC-based results are much higher than those stemming from an E-FGC model, for a fixed value of *μ*. Furthermore, a gradual increase in the buckling load is observed for an increased value of *μ*, independent of the selected model.

## 6. Conclusions

This paper presents a thermomechanical buckling analysis of sandwich beams with two identical CNTRC skins, under different hygrothermal environmental conditions and elastic foundation parameters, based on an E&P-FGC model. Different FGM theories are employed here in a unified framework for the first time, in conjunction with different CNTs’ agglomeration assumptions. By using the virtual displacement approach and Navier-type solution, we determine and solve the governing equations of the problem. Based on a large systematic investigation aimed at determining the thermomechanical buckling response and its sensitivity under different input parameters, some considerable conclusions can be summarized, as follows:

(a) The use of a P-FGC model obtains a higher stiffness and buckling load with respect to an E-FGC model.
(b) The presence of clusters or CNTs’ concentrated regions has a beneficial effect on the mechanical behavior of such models.
(c) TSDT and HSDT provide the highest and lowest values of dimensionless critical buckling load, respectively, where SSDT and ESDT always produce intermediate results.
(d) The presence of a hygrothermal environment delivers lower levels of stiffness and critical buckling load on the system, whose results appear reasonable and consistent from a physical standpoint.

## Figures and Tables

**Table 1:** Possible combinations of *μ* and *η*.

Case	Result
μ=0	No-agglomerated composite.
μ=1	The whole composite layer serves as a big agglomerated region.
η=0	There is no CNT inside the clusters.
η=1	All CNTs are agglomerated inside the clusters.
μ=η=1	Fully CNTs agglomerated composite.

**Table 2:** Comparison of the dimensionless critical buckling load of FGM beams as computed in the current study with respect to the literature [37] (×100).

*S*	*a/h* = 5				*a/h* = 10			
	EBB		TB		EBB		TB	
	Ref. [37]	Present	Ref. [37]	Present	Ref. [37]	Present	Ref [37]	Present
0	0.535	0.566	0.488	0.521	0.535	0.566	0.523	0.554
0.5	0.347	0.365	0.319	0.340	0.347	0.365	0.340	0.359
1	0.267	0.282	0.246	0.263	0.267	0.282	0.261	0.277
2	0.208	0.220	0.192	0.205	0.208	0.220	0.204	0.216
5	0.176	0.186	0.160	0.171	0.176	0.186	0.171	0.182
7	0.169	0.178	0.152	0.163	0.169	0.178	0.164	0.174
10	0.160	0.169	0.144	0.154	0.160	0.169	0.156	0.165

**Table 3:** FGC material properties [38].

Material	*ρ* (Kg/m^3^)	*ν*	*E* (GPa)	*β* (10^−3^/K)	*α* (10^−6^/K)	*θ* (10^−6^/K)
Al_2_O_3_	3800	0.3	380	1	8.3	8.3
ZrO_2_	3000	0.3	151	0	10	10
Si_3_N_4_	2370	0.24	322.27	0	5.87	5.87
Al	2707	0.3	70	440	24	24

**Table 4:** CNTRC skin material properties [17].

Material	*ρ* (Kg/m^3^)	*E* (GPa)	*β* (10^−4^/K)	α (10^−6^/K)	*K* (GPa)	*G* (GPa)
PMMA	1150	2.5	20	45	2.6	0.93
CNT	1400	5646.6	3	3.45	-	-

**Table 5:** Fundamental details of the model.

FGC Material	CNTRC Material	*h_c_* (mm)	*h_t_* = *h_b_* (mm)	*a* (mm)
Al & ZrO_2_	CNTs & PMMA	5	2	30
N*	*μ*	*η*	V*	*s*
= $\frac{Na^{2}}{h_{c}^{3}{Ε}_{m}}$	0.3	0.4	0.2	2
*H_b_* (wt%H_2_O)	*T_b_* (K)	*L*_1_ (GN/m^3^)	*L*_2_ (KN/m)	
0.1	300	5	50	

**Table 6:** Dimensionless critical bucking load for different beam theories, FGC model and CNTs’ volume fractions *µ*.

Theory	E-FGC			P-FGC		
	*μ* = 0.1	*μ* = 0.2	*μ* = 0.3	*μ* = 0.1	*μ* = 0.2	*μ* = 0.3
HSDT	0.3981	0.6916	0.8487	2.3511	2.6438	2.8015
SSDT	0.4017	0.6957	0.8528	2.3527	2.6458	2.8036
ESDT	0.4059	0.7005	0.8579	2.3547	2.4684	2.8064
TSDT	0.4163	0.7237	0.8897	2.3685	2.6702	2.8336

## Data Availability

Not applicable.
